# Editorial Comment: Targeting Heme in Sickle Cell disease: New Perspectives on Priapism Treatment

**DOI:** 10.1590/S1677-5538.IBJU.2024.9926

**Published:** 2025-01-10

**Authors:** Luciano A. Favorito

**Affiliations:** 1 Universidade do Estado do Rio de Janeiro Unidade de Pesquisa Urogenital Rio de Janeiro RJ Brasil Unidade de Pesquisa Urogenital - Universidade do Estado do Rio de Janeiro - Uerj, Rio de Janeiro, RJ, Brasil; 2 Hospital Federal da Lagoa Serviço de Urologia Rio de Janeiro RJ Brasil Serviço de Urologia, Hospital Federal da Lagoa, Rio de Janeiro, RJ, Brasil

Tammyris Helena Rebecchi Silveira 1, Fabiano Beraldi Calmasini 2, Mariana Gonçalves de Oliveira 1, Fernando Ferreira Costa 3, Fábio Henrique Silva 1

**Eur Urol Open Sci. 2024 Mar 6:62:91-98.**

10.3389/fphys.2024.1435220 | ACCESS: 39086934

## COMMENT

Ischemic priapism, also called low-flow or ischemic priapism, is associated with decreased venous return with vascular stasis, which causes tissue hypoxia. In this type of priapism, venous drainage is delayed. It is usually painful due to ischemia. Ischemic priapism is associated with a high risk of fibrosis of the corpora cavernosa and impotence (1). In this interesting narrative review from Brazil the authors shows some interesting aspects of the priapism treatment (2) and concluded that the dysfunction of the nitric oxide (NO) and cyclic guanosine monophosphate (cGMP) pathway in erectile tissues as a critical mechanism in developing priapism in Sickle cell disease. Pharmacological treatments should ideally target the pathophysiological basis of the disease. Agents that reduce excess free heme in the plasma have emerged as potential therapeutic candidates for priapism treatment.
